# Solubilities of Amino Acids in Aqueous Solutions of Chloride or Nitrate Salts of Divalent (Mg^2+^ or Ca^2+^) Cations

**DOI:** 10.1021/acs.jced.2c00148

**Published:** 2022-05-18

**Authors:** Mehriban Aliyeva, Paula Brandão, José R. B. Gomes, João A. P. Coutinho, Olga Ferreira, Simão P. Pinho

**Affiliations:** † Centro de Investigação de Montanha (CIMO), Instituto Politécnico de Bragança, Campus de Santa Apolónia, 5300-253 Bragança, Portugal; ‡ CICECO − Aveiro Institute of Materials, Department of Chemistry, University of Aveiro, 3810-193 Aveiro, Portugal

## Abstract

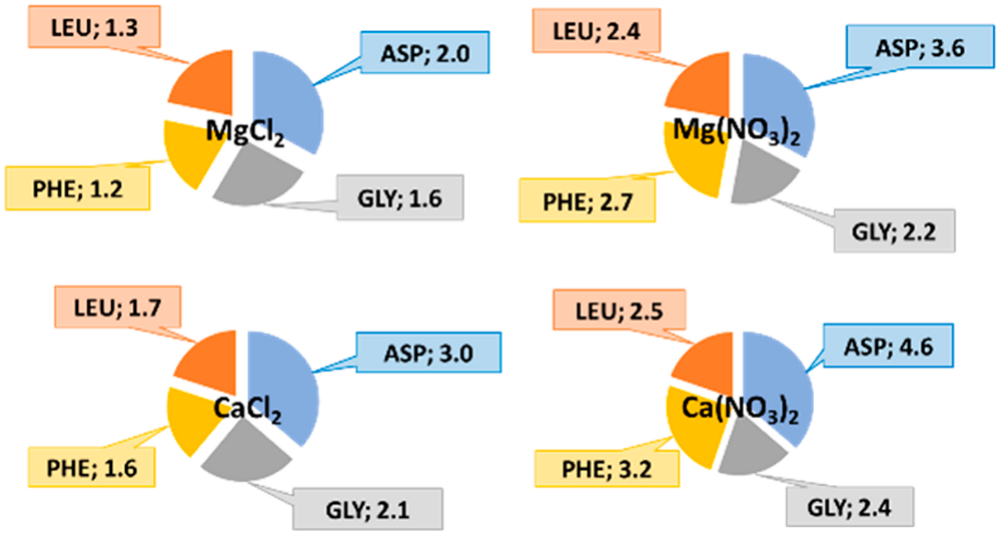

The solubilities of glycine, l-leucine, l-phenylalanine, and l-aspartic acid were measured in aqueous MgCl_2_, Mg(NO_3_)_2_, CaCl_2,_, and Ca(NO_3_)_2_ solutions with concentrations ranging from 0 to 2 mol/kg at 298.2 K. The isothermal analytical method was used combined with the refractive index measurements for composition analysis guaranteeing good accuracy. All salts induced a salting-in effect with a higher magnitude for those containing the Ca^2+^ cation. The nitrate anions also showed stronger binding with the amino acids, thus increasing their relative solubility more than the chloride anions. In particular, calcium nitrate induces an increase in the amino acid solubility from 2.4 (glycine) to 4.6 fold (l-aspartic acid) compared to the corresponding value in water. Amino acid solubility data in aqueous MgCl_2_ and CaCl_2_ solutions collected from the open literature were combined with that from this work, allowing us to analyze the relations between the amino acid structure and the salting-in magnitude.

## 1 Introduction

Knowledge of the solubility of biomolecules such as proteins in aqueous electrolyte solutions is central to understanding biochemical processes and controlling solution behavior in many scientific and industrial applications.^1,2^ In addition, solubility data in this kind of systems is helpful to study protein destabilization and precipitation, which is very important in the pharmaceutical field.^2−4^ Factors such as pH, ionic strength, temperature, and additives can change the solubility of proteins,^3−7^ and it has been noticed that the concentration and chemical characteristics of ions present can introduce multiple and significant effects that still need to be better and more deeply understood.^2,3,5,6,8−13^ Owing to the complexity of proteins,^13,14^ to simplify the difficulty of obtaining reliable and consistent quantitative solubility data,^4^ amino acids (AA) and small peptides can be used as model compounds to rationalize the salt effect on biomolecules solubility phenomena. Although many studies to understand the effect of salts on the AA solubility have been published, these are mainly concerned with systems containing monovalent ions,^15−21^ and a lack of data on the solubility of amino acids in aqueous electrolyte solutions containing divalent cations is still in demand.

Divalent metal cations such as Mn^2+^ and Zn^2+^ are important in many enzymatic reactions, Cu^2+^ and Fe^2+^ are essential ions of respiration and photosynthesis,^22^ the Ca^2+^ cation has been used in the solubilization of myofibrillar proteins with relevance in the food industry,^23^ and γ-glycine crystals produced from aqueous solutions with the Mg^2+^ cation were shown to be effective in laser applications and fabrication of electro-optical devices.^24^ Additionally, the relevance of Zn^2+^, Mg^2+^, and Ca^2+^ to stabilize the structure of folded proteins and, in some cases, to fix a particular physiologically active conformation of the protein is well established.^25^ Classical molecular dynamics (MD) simulations have also been carried out to understand the intermolecular interaction between the divalent or polyvalent cations with dipeptides^22^ and AA.^15^

Our previous work focused on sodium, potassium, and ammonium salts, combined with many different anions, presenting an extensive comparison for data consistency, and broader interpretation, by compiling solubility data from the open literature for a large set of AA and measuring the solubility of l-aspartic acid, l-phenylalanine, l-leucine, and glycine, in aqueous systems of chloride and nitrate salts with the above-mentioned cations.^26^ Contributing to fill the gap on systems with a divalent cation, in this work, the solubility measurement of the same four AA in aqueous solutions of MgCl_2_, Mg(NO_3_)_2_, CaCl_2_, or Ca(NO_3_)_2_ was carried out up to a salt molality of 2 mol/kg at 298.2 K. To the best of our knowledge, for this set of AA, only for glycine some solubility data have been published in aqueous solutions of MgCl_2_^17,19,20^ and CaCl_2_.^15,16,18^ While these works also present information on amino acids such as alanine, valine, isoleucine, or serine, no data were found for AA in aqueous Mg(NO_3_)_2_ and Ca(NO_3_)_2_ solutions.

**Figure 1:**
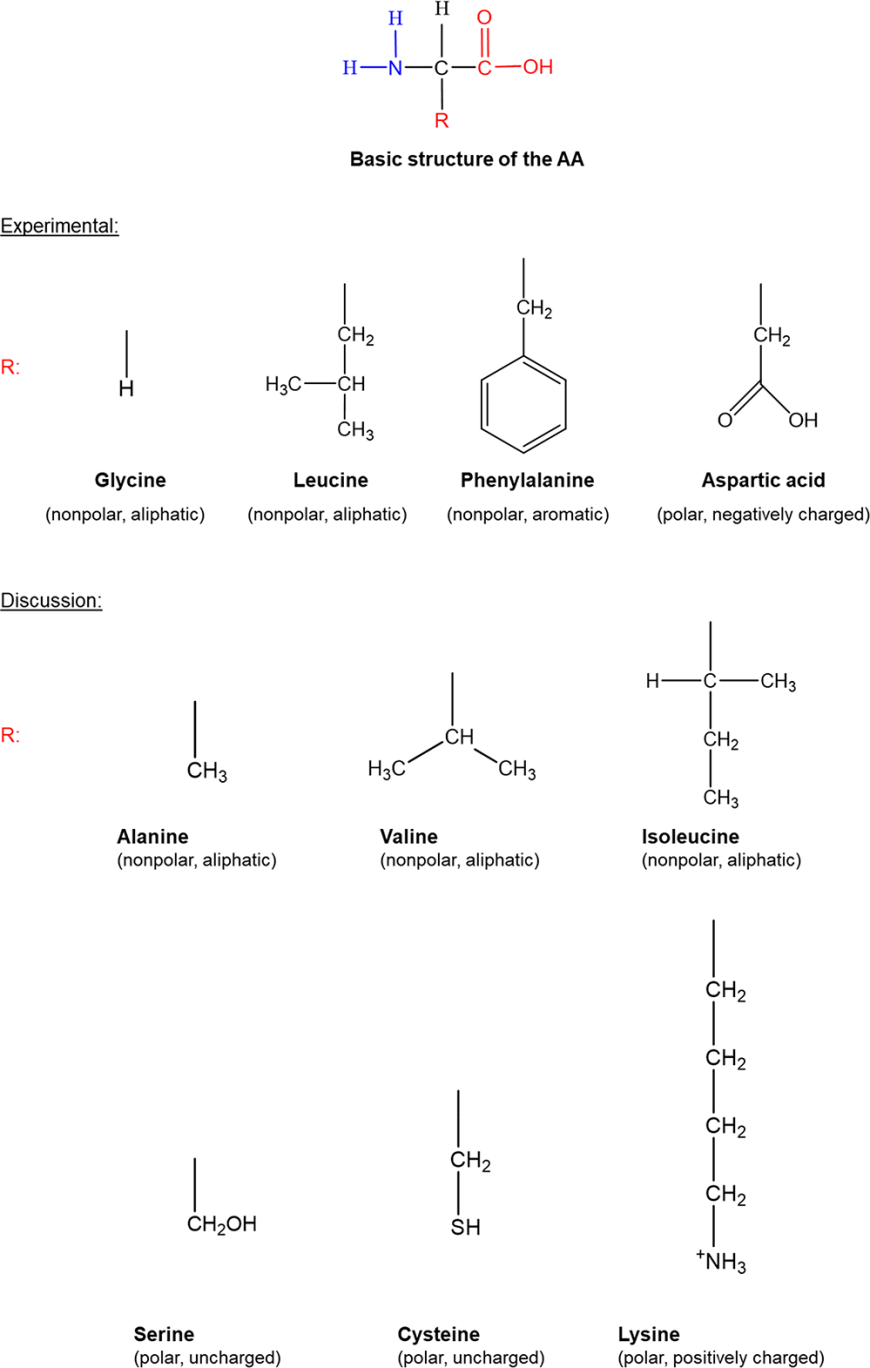
Basic structure and specific side chains of the AA. Experimental: for which solubility measurements were performed. Discussion: for which solubility data was collected from the literature.

The basic structure and specific side chain of the AA studied in this work are given in Figure 1 (side chain characterized in terms of polarity and charge at physiological pH). As can be seen in Figure 1, this work also contributes to filling the gap on data for AA containing an aromatic group and on those with more than one carboxylic acid group.

## 2 Experimental Section

### 2.1 Chemicals

The source, CAS number, and purity of the used chemicals are given in Table 1. All the AA were used without further purification and were stored in a desiccator to keep the AA dry. According to the certificate of analysis, their mass fraction purity is ≥0.98. The electrolyte solutions were prepared using deionized water (resistivity of 18.2 MΩ·cm, particles with size < 0.22 μm, and total organic carbon < 5 ppb).

**Table 1:** Name, Source, CAS Number, and Mass Fraction Purity of the Compounds Used

name	supplier	CAS	mass fraction purity
Glycine (Gly)	Merck	56-40-6	≥0.997
l-leucine (Leu)	Merck	61-90-5	≥0.990
l-phenylalanine (Phe)	Merck	63-91-2	≥0.990
l-aspartic acid (Asp)	Alfa Aesar	56-84-8	≥0.980
magnesium chloride hexahydrate	PanReac	7791-18-6	≥0.990
magnesium nitrate hexahydrate	PanReac	13446-18-9	≥0.980
calcium chloride dihydrate	Fluka	10035-04-8	≥0.990
calcium nitrate tetrahydrate	Alfa Aesar	13477-34-4	≥0.990

### 2.2 Solubility Experiments

For the solubility measurement, the isothermal analytical method was applied, and the AA concentration was found by measuring the refractive index of the saturated solutions. As the salts are all hydrated, the determination of their water content was first carried out by Karl Fischer (KF) titration, and that value was considered for the salt molality determinations. The water + salt solutions (0.5, 1, or 2 mol/kg) were prepared by weight (Denver Instrument, ± 0.0001 g). The calibration curve (*R*^2^ > 0.996, Figure S1), relating the amino acid concentration (in g kg^–1^ of solution) and the refractive index, was then built by weighting six standard solutions (Denver Instrument, ± 0.0001 g) of known AA composition at a fixed salt concentration. The refractive index was measured (at 298.2 K) in a digital refractometer (Abbemat 500, Anton Paar) with a reproducibility within ±0.00002.

To start the solubility measurements, the ternary solutions were prepared by adding an excess amount of the AA into the stoppered glass tubes and a known mass of solvent (water + salt). The contents of the tubes were stirred in the water bath for around 30 h to attain equilibrium, and the temperature was set at 298.2 K (±0.1 K). The speed of the magnetic stirrer was kept in the range of 500 to 700 rpm. After, the mixing was stopped for at least 12 h to ensure the undissolved particles settled at the bottom of the equilibrium cell. Four samples of the saturated solutions (approximately 2–3 cm^3^) were collected using preheated plastic syringes coupled with polypropylene filters (0.45 μm), placed into glass vessels, weighed, and if needed, diluted with a weighed amount of (water + salt) solution in order to get refractive indexes within the calibration curve range.

Finally, the refractive indexes of each solution were determined twice, and the solubility value was calculated from the calibration curve. At least four independent values are used to find the final average solubility. It is very important to note that the exactly same aqueous salt solution (0.5, 1, or 2 mol/kg) initially prepared is used to find the calibration curve, to prepare the saturated solution, and also to dilute (when needed) the saturated solution before the refractive index measurement.

### 2.3 Solid Phase Studies

The solid phase of the pure AA as received from the supplier and solids equilibrated with the saturated solutions, after vacuum filtration and drying at room temperature, were analyzed by powder and single-crystal X-ray diffraction.

Powder XRD data were collected on a X’Pert MPD Philips diffractometer, using Cu Ka radiation (λ = 1.5406 Å), with a curved graphite monochromator, a set incident area of 10 mm^2^, and a flat plate sample holder, in a Bragg–Brentano para-focusing optics configuration. Intensity data were collected by the step counting method (step 0.02° and time 5 s) in the range 5° < 2θ < 50°.

The cell parameters of suitable crystals of selected l-aspartic acid, l-phenylalanine, glycine, and l-leucine from supply as well the samples obtained after crystallization with the different salts, were determined on a Bruker D8 QUEST diffractometer equipped with a Photon 100 area detector, with monochromated Mo Kα radiation (λ = 0.71073 Å) and operating at 150(2) K. The selected crystals analyzed were put at 40 mm from the photon 100 detector, and the spots were measured using different counting times (varying from 5 to 30 s).

## 3 Results and Discussion

### 3.1 Experimental Data and Analysis

Table 2 compiles the measured solubilities along with the standard deviation (in brackets) of l-aspartic acid, l-phenylalanine, l-leucine, and glycine in the aqueous MgCl_2_, Mg(NO_3_)_2_, CaCl_2_, and Ca(NO_3_)_2_ solutions with a salt molality of 0, 0.5, 1.0, and 2.0 at 298.2 K. In all the studied systems the absolute solubility follows the order Gly > Phe > Leu > Asp, as in pure water.

**Table 2:** Solubility of the Amino Acids (g of AA/kg of Water, Standard Deviation in Brackets) in Aqueous Solutions of Salts at Different Molalities, *T* = 298.2 K and *p* = 0.1 MPa^a^

		*S*_AA_ (g of AA/1000 g of water)			
salts	electrolyte molality (*m*, mol/kg)	glycine	l-leucine	l-phenylalanine	l-aspartic acid
no salt	0.000	238.332* (0.127)	21.544* (0.070)	28.347* (0.083)	5.140* (0.031)
MgCl_2_	0.500	272.843 (0.143)	24.939 (0.082)	32.632 (0.043)	6.722 (0.051)
	1.000	293.517 (0.340)	24.465 (0.049)	33.012 (0.138)	7.917 (0.172)
	2.000	370.316 (0.394)	28.497 (0.119)	34.289 (0.202)	10.300 (0.143)
Mg(NO_3_)_2_	0.500	303.191 (0.168)	29.895 (0.076)	42.916 (0.066)	8.649 (0.051)
	1.000	355.417 (0.052)	36.981 (0.226)	54.307 (0.456)	11.443 (0.328)
	2.000	519.400 (0.153)	51.925 (0.146)	76.838 (0.184)	18.414(0.380)
CaCl_2_	0.500	294.982 (0.985)	26.275 (0.080)	34.812 (0.164)	8.421 (0.128)
	1.000	354.148 (0.700)	29.801 (0.064)	39.420 (0.767)	9.951 (0.077)
	2.000	489.824 (0.650)	35.734 (0.385)	44.668 (0.081)	15.497 (0.189)
Ca(NO_3_)_2_	0.500	316.463 (0.312)	30.768 (0.429)	44.111 (0.143)	9.492 (0.010)
	1.000	393.988 (0.378)	38.170 (0.169)	57.899 (0.352)	13.418 (0.470)
	2.000	578.196 (0.094)	54.255 (0.391)	90.694 (0.458)	23.830 (0.451)

a Published in Aliyeva et al.^26^ Standard uncertainties: *u* (*T*) = 0.10 K, *u*_r_ (*p*) = 0.05. Combined uncertainty: *u*_c_ (*m*) = 0.014 m.

The pH of each saturated solution was also measured at 298.2 K and the values listed in Table 3. As the pH values of the solutions are very close to the isoelectric point, it can be concluded that AA in the saturated solutions are in the zwitterionic form (dipolar ions), which was also confirmed by the AA speciation calculated in the Chemspider platform.^27,28^

**Table 3:** pH Values for the Different AA Saturated Solutions in Aqueous Electrolyte Solutions at 298.2 K^a^

	pH range in the ternary solution			
amino acids	MgCl_2_	Mg(NO_3_)_2_	CaCl_2_	Ca(NO_3_)_2_
glycine	5.56–5.97*	5.79–5.97*	5.91–5.97*	5.97*–6.04
leucine	5.72–5.98*	5.72–5.98*	5.94–5.98*	5.98*–6.04
phenylalanine	5.25–5.48*	5.39–5.48*	5.47–5.48*	5.48*–5.70
aspartic acid	2.55–2.77*	2.46–2.77*	2.48–2.77*	2.57–2.77*

a Saturated solution in water.^29^*u* (pH) = 0.05, *u* (*T*) = 0.15 K.

The solids from the supplier were analyzed by single crystal and powder diffraction. Glycine from supply presents a mixture of two phases, a monoclinic corresponding to the α-form and a hexagonal corresponding to γ-form (Figure S2 and Table S1), both already described in the literature. All the other amino acids from the supplier show a single phase, and are also well characterized in the literature. Table S1 of SI presents the structure, cell parameters, CCDC code, and references relative to the crystal forms found. In all four aqueous electrolyte solutions glycine solid phase (Figure S3) is only in the hexagonal crystal system, the γ-form. Generally, the crystalline form of the other amino acids (Figures S4, S5, and S6) in equilibrium with the saturated solutions containing the different electrolytes does not change compared to the structure found in the solid from the supplier. The only exception is for phenylalanine in aqueous solutions of MgNO_3_, containing a second phase. Searching in the ICDD database (version 2022), it was impossible to identify this second phase.

**Figure 2:**
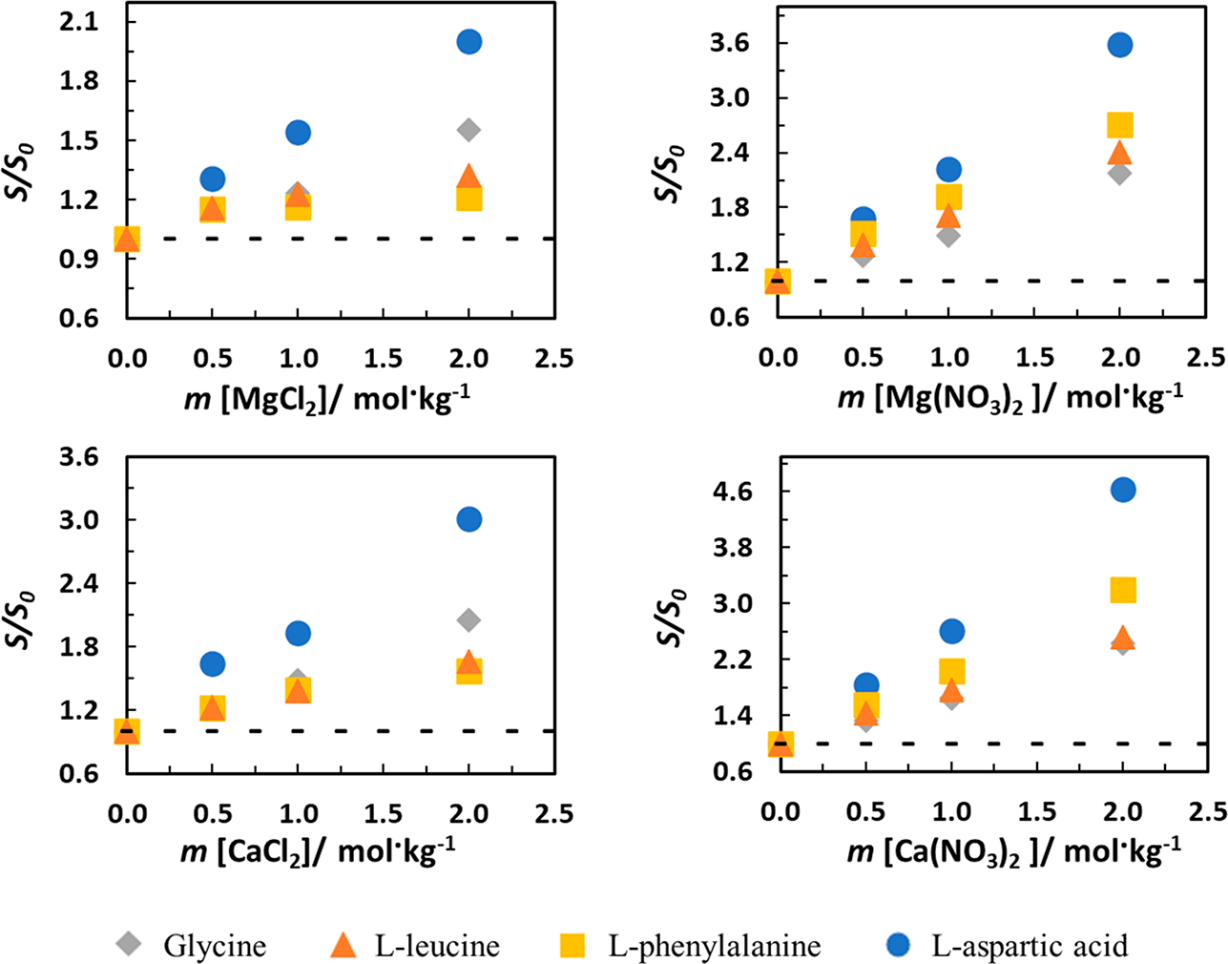
Relative solubility (*S*/*S*_0_) of AAs in aqueous salt solutions with different molalities at 298.2 K.

Figure 2 presents the relative solubilities (ratio between the solubility of AA (*S*), expressed as the mass of the amino acid in 1 kg of water, in aqueous salt solutions to that in pure water (*S*_0_) of glycine, l-leucine, l-phenylalanine, and l-aspartic acid in aqueous MgCl_2_, Mg(NO_3_)_2_, CaCl_2_, and Ca(NO_3_)_2_ solutions. All the salts with divalent cations induce a salting-in effect over the whole salt concentration range for all the AA. l-aspartic acid, the most polar AA, due to the presence of a second negatively charged hydrophilic carboxyl group (−CH_2_COO^–^) in the side chain, presents the highest salting-in magnitude in all the aqueous salt solutions studied, which can attain a relative solubility of 4.6 in a 2 mol/kg calcium nitrate aqueous solution. The ranking differs mostly with the anion type, the relative solubilities in aqueous MgCl_2_ and CaCl_2_ solutions follow the order Asp > Gly > Leu ≅ Phe, while in the aqueous Mg(NO_3_)_2_ and Ca(NO_3_)_2_ solutions, the ranking is Asp > Phe > Leu ≅ Gly. The main difference is connected to the relative position of Phe and Gly, which confirms that nitrate anions are more effective in interacting with the apolar moieties of the AA, increasing the solubility of phenylalanine, and even leucine, with a larger apolar side chain, when compared to glycine.

**Figure 3:**
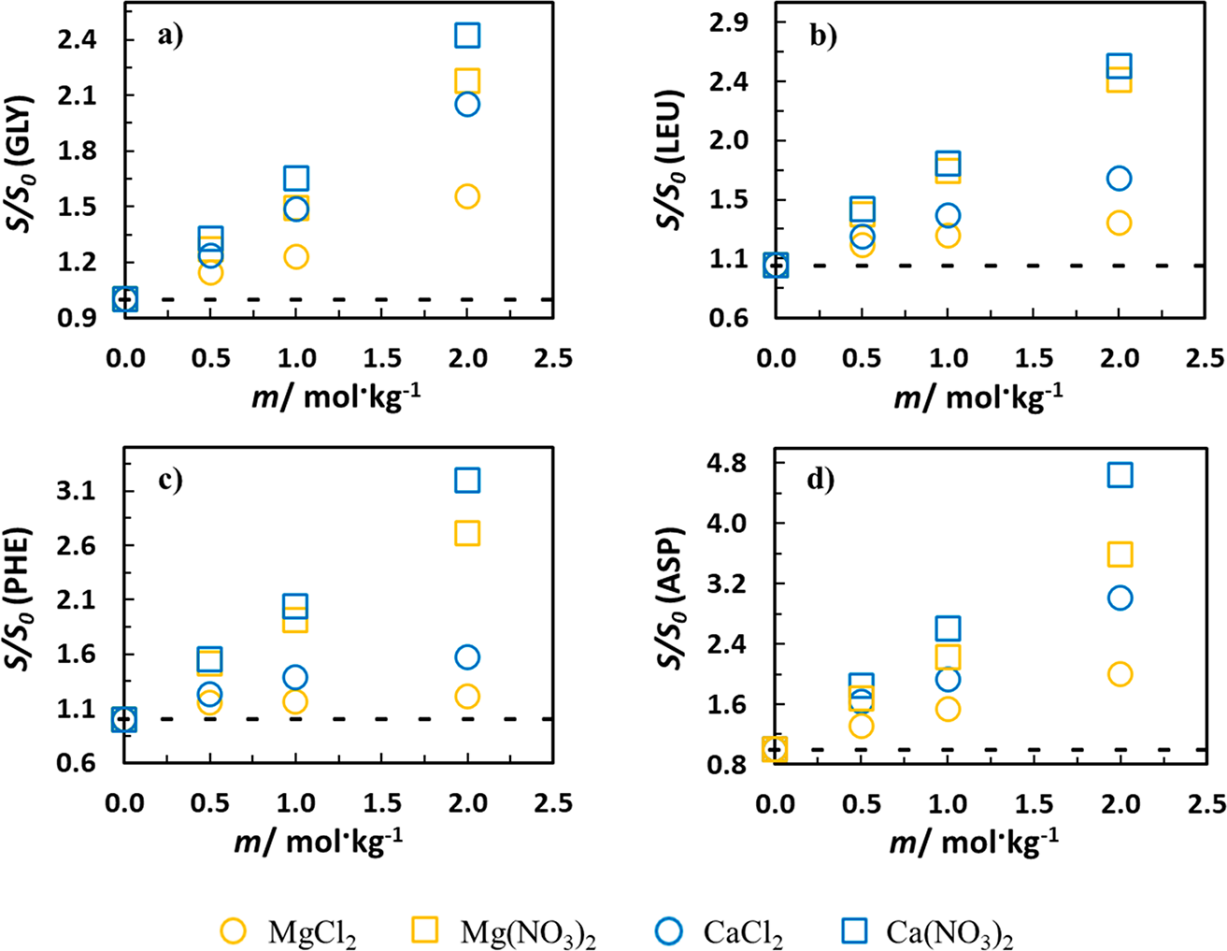
Relative solubility (*S*/*S*_0_) of a) glycine; b) l-leucine; c) l-phenylalanine, and d) l-aspartic acid in aqueous salt solutions with different salt molalities at 298.2 K.

Figure 3 shows the effect of all the studied salts on the relative solubility of each amino acid. As observed previously in the study with the monovalent cations,^26^ comparing salt solutions with the same divalent cation, the effect of nitrate anion on the relative solubilities of all the AA is higher than with chloride anions. In fact, using MD simulations, Tomé et al.^30^ showed that interaction of the NO_3_^–^ anion with the hydrophobic groups of the AA is more significant than with chloride, causing a larger solubility increase. In the Hofmeister series,^31^ these anions are close to each other, but the nitrate anion is more to the right side, that is, to where salting-in anions are located. Maintaining the anion and changing the cation, the Ca^2+^ cation induces a salting-in effect with a higher magnitude than the Mg^2+^, again in consistency with the Hoffmeister series where Ca^2+^ is the strongest salting-in cation. MD in systems with isoleucine as model AA showed very strong binding of the polyvalent cations to the carboxylate (COO)^−^ group of the amino acid. As demonstrated in several works,^25,32,33^ this type of interaction leads to the formation of stable complexes between the biomolecules and the divalent cations. However, the magnitude of the peaks in the radial distribution functions, or the distance of its appearance, is not totally conclusive since the Ca^2+^ cation presents very similar values compared to Mg^2+^. Nevertheless, both divalent cations present much stronger interaction than monovalent cations, but in both cases, interactions with the hydrophobic parts of the AA are not significant.

Globally, glycine shows a salting-in effect with close magnitudes in Ca(NO_3_)_2_, Mg(NO_3_)_2_, and CaCl_2_ solutions, that magnitude being much lower in aqueous MgCl_2_ solution (evaluated at 2 mol/kg). l-Leucine and l-phenylalanine present a salting-in effect with similar magnitudes in both nitrate solutions and much lower, even if with similar magnitudes, in the solutions with the chloride anions. All the salts induce a salting-in effect in l-aspartic acid, being, among the studied AA, the one showing the larger change in solubility induced by all four salts studied.

**Figure 4:**
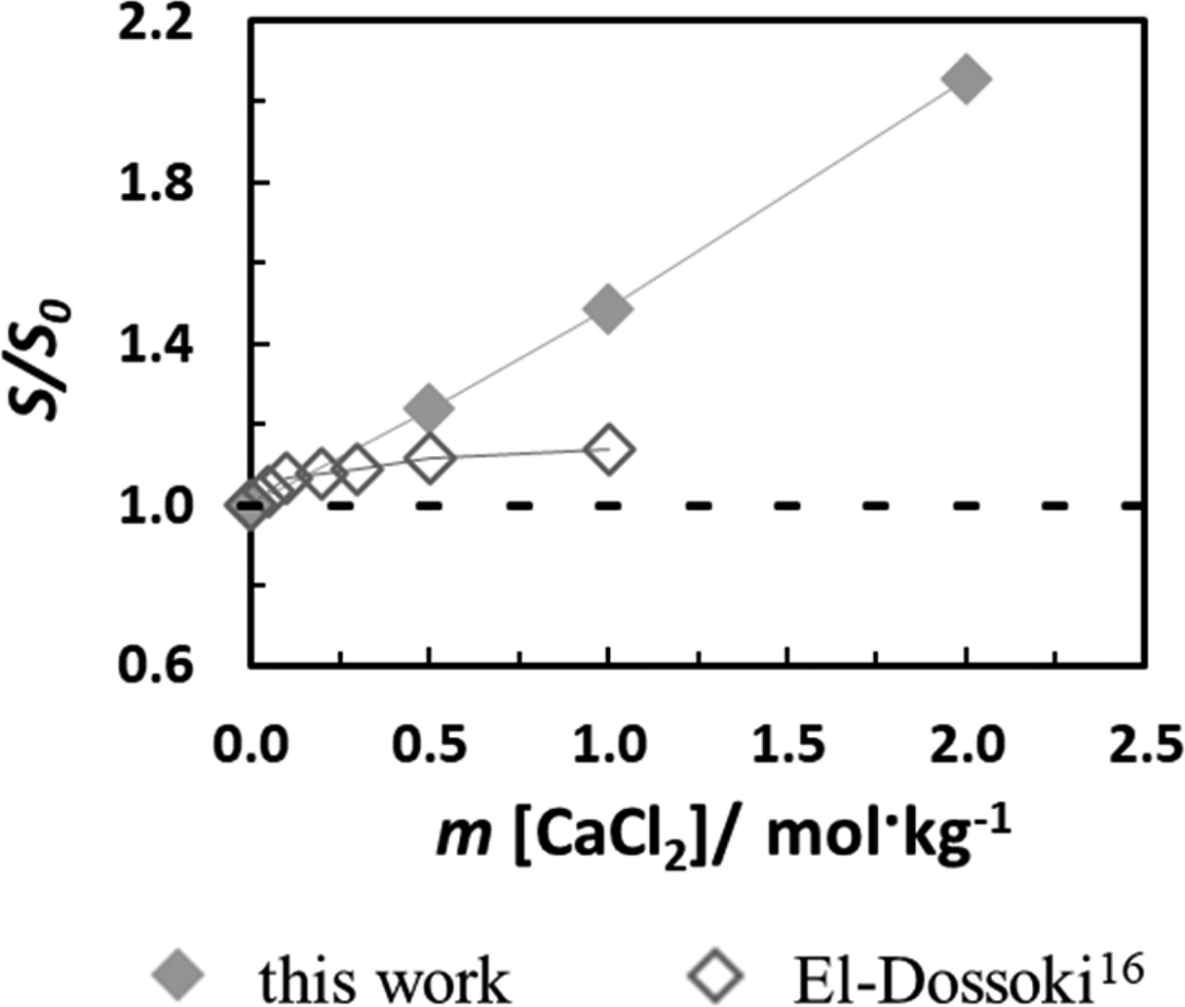
Relative solubility (*S*/*S*_0_) of glycine in aqueous CaCl_2_ solution at 298.2 K. Lines are a guide to the eyes.

For l-leucine, l-phenylalanine, and l-aspartic acid, no data in the studied aqueous salt solutions were found in the literature, and no comparisons can be presented. The solubility of Gly was studied in aqueous MgCl_2_ solutions,^17,19^ but not at 298.2 K. However, in the temperature range between 293.2 and 303.2 K, in a two mol/kg MgCl_2_ solution the relative solubility is close to 1.6,^17^ while in this work at 298.2 K the corresponding value is close to 1.55, showing good coherence between both works. As shown in Figure 4, a comparison can be made for the relative solubility of Gly in aqueous CaCl_2_ solutions. In both works, a salting-in effect is observed, but the magnitude differs significantly at higher concentrations. A similar situation was also reported by Tomé et al.^15^ for dl-alanine in an aqueous CaCl_2_ solution. As discussed in the previous work on monovalent cations,^26^ and by Tomé et al.,^15^ the data from El-Dossoki^16^ need to be carefully checked as multiple significant discrepancies have been found.

**Figure 5:**
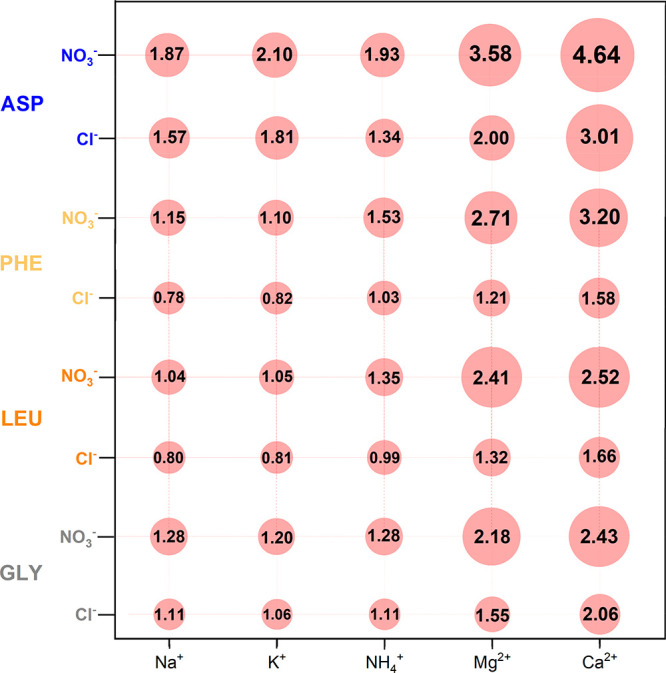
Relative solubility (*S*/*S*_0_) of glycine, l-leucine, l-phenylalanine, and l-aspartic acid in 2 mol/kg aqueous solutions of MgCl_2_, Mg(NO_3_)_2_, CaCl_2_, Ca(NO_3_)_2_, NaCl,^26^ NaNO_3_,^26^ KCl,^26^ KNO_3_,^26^ NH_4_Cl,^26^ and NH_4_NO_3_^26^ at 298.2 K.

Figure 5 shows the relative solubility of glycine, l-leucine, l-phenylalanine, and l-aspartic acid in 2 mol/kg aqueous solutions of the salts combining one of the Na^+^, K^+^, NH_4_^+^, Mg^2+^, Mg^2+^ cations with the Cl^–^ or NO_3_^–^ anion, at 298.2 K. A comparison of the effect of the salts with the same anion shows that the divalent cations induce a much higher salting-in effect than the salts with the monovalent cations for all AA. In the case of Gly, both salts with the divalent cations and the chloride anion show higher relative solubility than those with the monovalent cations and the nitrate anions. The apolar moiety in Gly is very small, and the balance is more favorable for the interaction of the divalent cation with the carboxylate if compared to the interaction between the nitrate and Gly. For the rest of the AA, the order differs. Accordingly, the results in the solution with l-leucine and NH_4_NO_3_ show a salting-in effect of the same magnitude as MgCl_2_, while in l-phenylalanine, with an apolar aromatic side chain, the salting-in effect of NH_4_NO_3_ is comparable to that observed in aqueous CaCl_2_ solutions (with the strongest salting-in cation). This is a consequence of the nitrate anion interaction^30^ with the apolar AA moieties leading to a more relevant salting-in effect. Comparing divalent cations, the salting-in effect enhancement when moving from chloride to nitrate is highest in l-phe, similar for l-leu and l-asp, and the lowest in gly.

Additionally, that enhancement is more significant in magnesium than calcium salts. When fixing the anion, the salting-in change, moving from magnesium to calcium salts, is more evident in chlorides, while monovalent to divalent cations are more significant in nitrates. Despite its small apolar region, l-asp is the only AA showing a similar salting-in effect in aqueous MgCl_2_ as in all nitrate solutions of monovalent cations.

### 3.2 Effect of the AA Structure

**Figure 6:**
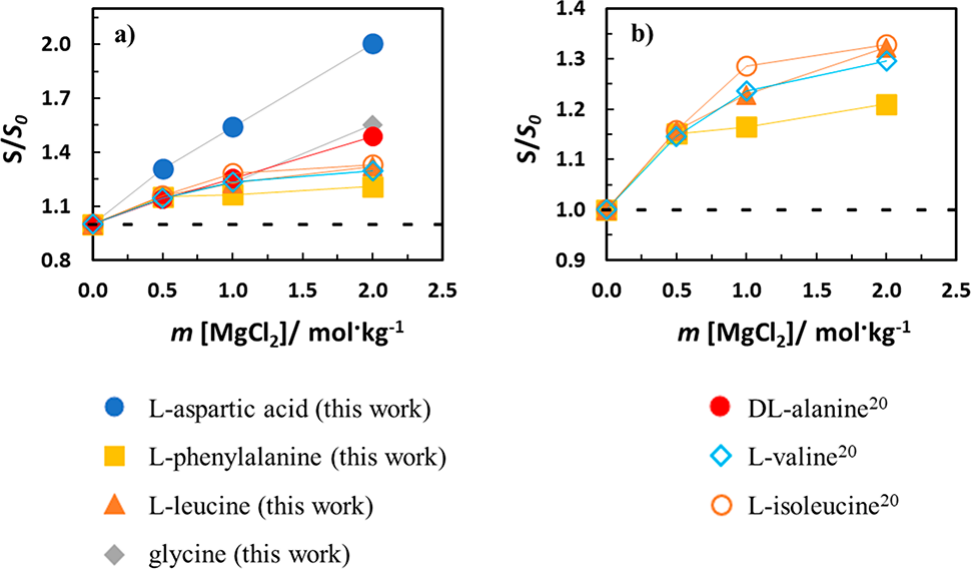
Relative solubility (*S*/*S*_0_) of different AAs in aqueous MgCl_2_ solution at 298.2 K. Lines are a guide to the eyes.

The effect of the AA side chain was studied by collecting data from different AA in aqueous solutions of chloride salts, found in the open literature, and analyzed all together with those measured in this work. For aqueous MgCl_2_ solutions, results were found just for three AA (dl-alanine, l-valine, and l-isoleucine).^20^Figure 6 presents the relative solubility change in aqueous MgCl_2_ solutions, showing a salting-in effect with the seven AA. l-aspartic acid with the polar acidic side chain shows the highest salting-in, followed by glycine with just hydrogen as the side chain, followed by dl-alanine, which has an additional methyl group, compared to glycine. As the AA with larger apolar groups show a very similar salting-in effect, Figure 6b is presented to understand the AA ranking more easily. The branched-chain aliphatic AA, l-leucine and l-isoleucine isomers differing slightly in their chemical structure, are followed by l-valine, which has one methylene group less than leucine or isoleucine but is bulkier. This is consistent with having, among all AA studied, l-phenylalanine as the one presenting the lowest salting-in effect.

**Figure 7:**
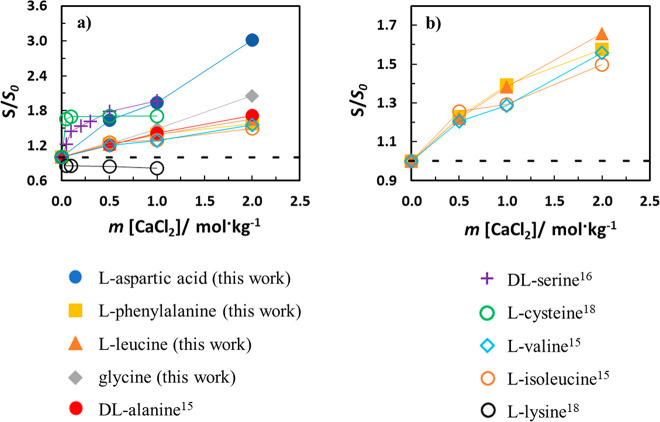
Relative solubility (*S*/*S*_0_) of different AAs in aqueous CaCl_2_ solution at 298.2 K. Lines are a guide to the eyes.

Figure 7 shows a similar comparison in aqueous CaCl_2_ solutions. This salt induces a salting-out effect just for l-lysine.^18^ Lysine is an alkaline, aliphatic AA whose side chain l-lysine ((CH_2_)_4_NH_2_) contains one extra amino group. This demonstrates that besides the weak interaction of chloride anion with the alkyl moieties of the molecule, the low interaction of chloride anion with the amine group of the AA is also revealed. This is surprising as the strong interaction of Ca^2+^ with the carboxylate is somehow lost by the presence of a large hydrophobic group, which did not happen with isoleucine, for instance. However, the data seem unreliable, also due to the very close salting-out magnitudes caused by NaCl or CaCl_2_, as reported in the work of El-Dossoki.^18^ The presence of two −COOH groups in l-aspartic acid and the polar, hydrophilic −OH group in dl-serine (increases the polarity of its hydrocarbon side chain) leads to a more pronounced salting-in effect. The thiol side chain (−SH) of l-cysteine seems to be a reason for the very pronounced salting-in at the salt infinite dilution, bringing some doubts again on data reliability as no changes in the solubility are observed at higher salt molalities. In terms of the AA with a completely apolar side chain, after glycine, salting-in decreases in the order dl-alanine (R = −CH_3_), l-leucine (R = −C_4_H_9_), l-phenylalanine (R = −CH_2_C_6_H_5_), l-valine (R = −C_3_H_7_), and l-isoleucine (R = −C_4_H_9_) showing a salting-in effect with very close magnitudes. In aqueous MgCl_2_ solution the relative solubility order of AA with apolar side chains follows: dl-ala > l-leu > l-ile > l-val > l-phe while in CaCl_2_: dl-ala > l-leu > l-phe > l-ile. All the AA studied in this work and dl-alanine, l-isoleucine, and l-valine show salting-in effects with higher magnitudes in aqueous CaCl_2_ solutions.

## 4 Conclusions

The solubilities of glycine, l-leucine, l-phenylalanine, and l-aspartic acid were studied in MgCl_2_, Mg(NO_3_)_2_, CaCl_2_, and Ca(NO_3_)_2_ solutions at 298.2 K. The measurements were chosen to be provided at various concentrations of the salts, from 0 to 2 mol/kg. The salts of divalent cations induced a salting-in effect with all the studied AA. The relative solubility followed different rankings; in aqueous salt solutions with the chloride anion, the salting-in order is Asp > Gly > Leu ≅ Phe, while that with the nitrate anion is Asp > Phe > Leu ≅ Gly. All the AA showed a salting-in with a higher magnitude in aqueous salt solutions with the Ca^2+^ than with the Mg^2+^ cation. Both cation and anion effects are in agreement with the Hofmeister series.

The results presented in aqueous solutions of salts containing divalent cations were compared to those of salt solutions consisting of the monovalent cations with the same anions. Also noted was a complex interplay between the significant divalent cation and carboxylate interactions and that between the nitrate and the apolar moieties of the AA, making, for instance, ammonium nitrate as effective as calcium chloride in the increase of l-phe solubility. In terms of the relative impact on increasing AA solubility, generally magnesium is more significant than calcium salts, but when the anion is fixed, the salting-in magnitude changes, moving from magnesium to calcium salts. This is more evident in chlorides, but the change from monovalent to divalent cations is more significant in nitrates.

To elucidate the role of the side chain functional groups, a database on the solubility of AA in aqueous MgCl_2_ and CaCl_2_ solutions was compiled. An intriguing observation is a difference in the effect of an aromatic or aliphatic side chain on the relative solubility, which is different in aqueous solutions of MgCl_2_ or CaCl_2_. At this stage, it is relevant to reinforce the need to assess the reliability of the published solubility data carefully and increase the amount of consistent data diversifying the ions and AA.

## Supporting Information Available

Calibration curve example for amino acid concentration determination by refractive index; amino acid crystal form and cell parameters; diffractograms of pure amino acid crystals and solid phases precipitated from saturated solutions containing the salts (PDF)

## References

[ref1] Zhang R.; Zhou R.; Pan W.; Lin W.; Zhang X.; Li M.; Li J.; Niu F.; Li A. Salting-in Effect on Muscle Protein Extracted from Giant Squid (Dosidicus Gigas). Food Chem. 2017, 215, 256–262. 10.1016/j.foodchem.2016.07.177. 27542474

[ref2] Roberts D.; Keeling R.; Tracka M.; van der Walle C. F.; Uddin S.; Warwicker J.; Curtis R. Specific Ion and Buffer Effects on Protein-Protein Interactions of a Monoclonal Antibody. Mol. Pharmaceutics 2015, 12, 179–193. 10.1021/mp500533c. 25389571

[ref3] Chi E. Y.; Krishnan S.; Randolph T. W.; Carpenter J. F. Physical Stability of Proteins in Aqueous Solution: Mechanism and Driving Forces in Nonnative Protein Aggregation. Pharm. Res. 2003, 20, 1325–1336. 10.1023/A:1025771421906. 14567625

[ref4] Kramer R. M.; Shende V. R.; Motl N.; Pace C. N.; Scholtz J. M. Toward a Molecular Understanding of Protein Solubility: Increased Negative Surface Charge Correlates with Increased Solubility. Biophys. J. 2012, 102, 1907–1915. 10.1016/j.bpj.2012.01.060. 22768947 PMC3328702

[ref5] Wu L.; Wu T.; Wu J.; Chang R.; Lan X.; Wei K.; Jia X. Effects of Cations on the “Salt in” of Myofibrillar Proteins. Food Hydrocoll. 2016, 58, 179–183. 10.1016/j.foodhyd.2016.02.028.

[ref6] Nahar M. K.; Zakaria Z.; Hashim U.; Bari M. F. Effect of PH and Salt Concentration on Protein Solubility of Slaughtered and Non-Slaughtered Broiler Chicken Meat. Sains Malays 2017, 46, 719–724. 10.17576/jsm-2017-4605-06.

[ref7] Sathe S. K.; Zaffran V. D.; Gupta S.; Li T. Protein Solubilization. J. Am. Oil Chem. Soc. 2018, 95, 883–901. 10.1002/aocs.12058.

[ref8] Melander W.; Horvath C. Salt Effects on Hydrophobic Interactions in Precipitation and Chromatography of Proteins: An Interpretation of the Lyotropic Series’. Arch. Biochem. Biophys. 1977, 183, 200–215. 10.1016/0003-9861(77)90434-9. 907351

[ref9] Andreetta-Gorelkina I. V.; Greiff K.; Rustad T.; Aursand I. G. Reduction of Salt in Haddock Mince: Effect of Different Salts on the Solubility of Proteins. J. Aquat. Food Prod. Technol. 2016, 25, 518–530. 10.1080/10498850.2013.879241.

[ref10] Kalyuzhnyi Y. v.; Vlachy V. Explicit-Water Theory for the Salt-Specific Effects and Hofmeister Series in Protein Solutions. J. Chem. Phys. 2016, 144, 215101 10.1063/1.4953067. 27276970 PMC4902819

[ref11] Murakami S.; Hayashi T.; Kinoshita M. Effects of Salt or Cosolvent Addition on Solubility of a Hydrophobic Solute in Water: Relevance to Those on Thermal Stability of a Protein. J. Chem. Phys. 2017, 146, 055102 10.1063/1.4975165. 28178788

[ref12] Zhou H. X. Interactions of Macromolecules with Salt Ions: An Electrostatic Theory for the Hofmeister Effect. Proteins: Struct. Funct. Genet. 2005, 61, 69–78. 10.1002/prot.20500. 16044460

[ref13] Nandi P. K.; Robinson D. R. The Effects of Salts on the Free Energy of the Peptide Group. J. Am. Chem. Soc. 1972, 94, 1299–1307. 10.1021/ja00759a042. 5060273

[ref14] Balos V.; Kim H.; Bonn M.; Hunger J. Dissecting Hofmeister Effects: Direct Anion–Amide Interactions Are Weaker than Cation–Amide Binding. Angew. Chem. 2016, 128, 8257–8261. 10.1002/ange.201602769. 27237055

[ref15] Tomé L. I. N.; Sousa C. S. R.; Gomes J. R. B.; Ferreira O.; Coutinho J. A. P.; Pinho S. P. Understanding the Cation Specific Effects on the Aqueous Solubility of Amino Acids: From Mono to Polyvalent Cations. RSC Adv. 2015, 5, 15024–15034. 10.1039/C5RA00501A.

[ref16] El-Dossoki F. I. Effect of the Charge and the Nature of Both Cations and Anions on the Solubility of Zwitterionic Amino Acids, Measurements and Modeling. J. Sol. Chem. 2010, 39, 1311–1326. 10.1007/s10953-010-9580-3.

[ref17] Ansari Z. H.; Li Z. Solubilities and Modeling of Glycine in Mixed NaCl-MgCl2 Solutions in a Highly Concentrated Region. J. Chem. Eng. Data 2016, 61, 3488–3497. 10.1021/acs.jced.6b00403.

[ref18] El-Dossoki F. I.; El-Damarany M. M. Solvation of Basic and Neutral Amino Acids in Aqueous Electrolytic Solutions: Measurements and Modeling. J. Chem. Eng. Data 2015, 60, 2989–2999. 10.1021/acs.jced.5b00393.

[ref19] Ansari Z. H.; Zeng Y.; Zhang Y.; Demopoulos G. P.; Li Z. Modeling of Glycine Solubility in Aqueous HCl–MgCl2 System and Its Application in Phase Transition of Glycine by Changing Media and Supersaturation. J. Cryst. Growth 2017, 467, 116–125. 10.1016/j.jcrysgro.2017.03.037.

[ref20] Tome L. I. N.; Pinho S. P.; Jorge M.; Gomes J. R. B.; Coutinho J. A. P. Salting-in with a Salting-out Agent: Explaining the Cation Specific Effects on the Aqueous Solubility of Amino Acids. J. Phys. Chem. B 2013, 117, 6116–6128. 10.1021/jp4021307. 23638911

[ref21] Shi G.; Dang Y.; Pan T.; Liu X.; Liu H.; Li S.; Zhang L.; Zhao H.; Li S.; Han J.; Tai R.; Zhu Y.; Li J.; Ji Q.; Mole R. A.; Yu D.; Fang H. Unexpectedly Enhanced Solubility of Aromatic Amino Acids and Peptides in an Aqueous Solution of Divalent Transition-Metal Cations. Phys. Rev. Lett. 2016, 117, 1–6. 10.1103/PhysRevLett.117.238102. 27982649

[ref22] Santosh M. S.; Lyubartsev A. P.; Mirzoev A. A.; Bhat D. K. Molecular Dynamics Investigation of Dipeptide- Transition Metal Salts in Aqueous Solutions. J. Phys. Chem. B 2010, 114, 16632–16640. 10.1021/jp108376j. 21086976

[ref23] Wang Y.; Zhou Y.; Li P.-j.; Wang X.-x.; Cai K.-z.; Chen C.-g. Combined Effect of CaCl2 and High Pressure Processing on the Solubility of Chicken Breast Myofibrillar Proteins under Sodium-Reduced Conditions. Food Chem. 2018, 269, 236–243. 10.1016/j.foodchem.2018.06.107. 30100429

[ref24] Dillip G. R.; Bhagavannarayana G.; Raghavaiah P.; Deva Prasad Raju B. Effect of Magnesium Chloride on Growth, Crystalline Perfection, Structural, Optical, Thermal and NLO Behavior of γ-Glycine Crystals. Mater. Chem. Phys. 2012, 134, 371–376. 10.1016/j.matchemphys.2012.03.004.

[ref25] Dudev T.; Lim C. Principles Governing Mg, Ca, and Zn Binding and Selectivity in Proteins. Chem. Rev. 2003, 103, 773–787. 10.1021/cr020467n. 12630852

[ref26] Aliyeva M.; Brandão P.; Gomes J. R. B.; Coutinho J. A. P.; Ferreira O.; Pinho S. P. Electrolyte Effects on the Amino Acid Solubility in Water: Solubilities of Glycine, L-Leucine, L-Phenylalanine, and L-Aspartic Acid in Salt Solutions of (Na+, K+, NH4+)/(Cl-, NO3-). Ind. Eng. Chem. Res. 2022, 61, 5620–5631. 10.1021/acs.iecr.1c04562.

[ref27] Pence H. E.; Williams A. Chemspider: An Online Chemical Information Resource. J. Chem. Educ. 2010, 87, 1123–1124. 10.1021/ed100697w.

[ref28] Chemspider. http://www.chemspider.com/ (accessed 2021–05–30).

[ref29] Nelson D. L.; Cox M. M. Lehninger Principles of Biochemistry, 4th ed.; W. H. Freeman and Company: New York, 2005.

[ref30] Tomé L. I. N.; Jorge M.; Gomes J. R. B.; Coutinho J. A. P. Toward an Understanding of the Aqueous Solubility of Amino Acids in the Presence of Salts: A Molecular Dynamics Simulation Study. J. Phys. Chem. B 2010, 114, 16450–16459. 10.1021/jp104626w. 21090610

[ref31] Kunz W. Specific Ion Effects in Colloidal and Biological Systems. Curr. Opin. Colloid Interface Sci. 2010, 15, 34–39. 10.1016/j.cocis.2009.11.008.

[ref32] Dudev T.; Lim C. Effect of Carboxylate-Binding Mode on Metal Binding/Selectivity and Function in Proteins. Acc. Chem. Res. 2007, 40, 85–93. 10.1021/ar068181i. 17226948

[ref33] Tian J.; Yin Y.; Sun H.; Luo X. Magnesium Chloride: An Efficient 13C NMR Relaxation Agent for Amino Acids and Some Carboxylic Acids. J. Magn. Reson. 2002, 159, 137–144. 10.1016/S1090-7807(02)00016-2. 12482691

