# Supplementary material for: Solubilities of Amino Acids in Aqueous Solutions of Chloride or Nitrate Salts of Divalent (Mg2+ or Ca2+) Cations
Source: J Chem Eng Data. 2022 May 18;67(6):1565–72. doi: 10.1021/acs.jced.2c00148 (PMC9777878; doi:10.1021/acs.jced.2c00148)
Supplement: Supplementary file 1 — je2c00148_si_001.pdf [file je2c00148_si_001.pdf]

Supporting information

Solubilities of Amino Acids in Aqueous Solutions of Chloride or Nitrate Salts of Divalent (Mg^{2+} or Ca^{2+}) Cations

Mehriban Aliyeva[1,2], Paula Brandão[2], José R. B. Gomes[2], João A. P. Coutinho[2], Olga Ferreira[1,*], Simão P. Pinho[1,*]

[1]Centro de Investigação de Montanha (CIMO), Instituto Politécnico de Bragança, Campus de Santa Apolónia, 5300-253 Bragança, Portugal

[2]CICECO – Aveiro Institute of Materials, Department of Chemistry, University of Aveiro, 3810-193 Aveiro, Portugal

[*]To whom correspondence should be addressed:

Olga Ferreira, Telephone: +351273303087, Fax: +351273313051, E-mail address: oferreira@ipb.pt

Simão P. Pinho, Telephone: +351273303086, Fax: +351273313051, E-mail: spinho@ipb.pt

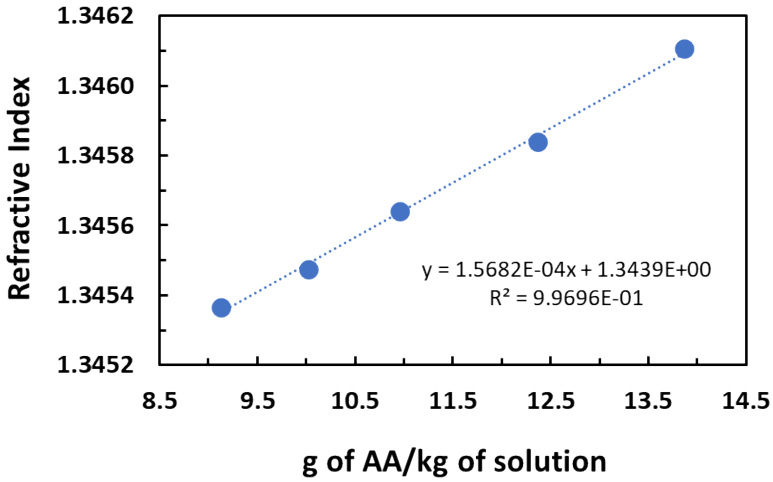

Figure S1. Calibration curve for L-leucine in aqueous $Ca(NO_3)_2$ solution at 0.5 molal.

Table S1. Cell parameters for glycine, L-aspartic acid, L-phenylalanine and L-leucine from supplier determined by single crystal X-ray diffraction and comparison with published data in CCDC Cambridge database.

Amino acid	Crystal form	CCDC code	Reference
Glycine	Monoclinic P (α-glycine) a=5.10 Å; b=11.99 Å; c=5.45 Å; β= 111.53°	1416373	Jiang *et al.*[1]
	Hexagonal P (γ-glycine) a=b=7.00 Å; c=5.48 Å	1416374	Jiang *et al.*[1]
L-Aspartic acid	Monoclinic P a=5.11 Å; b=6.92 Å; c=7.60 Å; β= 100.38°	652520	Bendeif and Jelsch[2]
L-Phenylalanine	Monoclinic P a=8.80 Å; b=6.06 Å; c=31.38 Å; β= 97.36°	1012155	Ihlefeldt *et al.*[3]
L-Leucine	Monoclinic P a=9.63 Å; b=5.34 Å; c=14.65 Å; β= 94.09°	1508364	Binns *et al.*[4]

Table S2. Cell parameters for amino acids in aqueous solutions of chloride or nitrate salts of divalent (Mg^{2+} or Ca^{2+}) Cations determined by single crystal X-ray diffraction and comparison with published data in CCDC Cambridge database.

Sample	Crystal form	CCDC code	Reference
Gly-$CaCl_2$<br>Gly-$CaNO_3$<br>Gly-$MgNO_3$<br>Gly-$MgCl_2$	Hexagonal P (γ-glycine)<br>a=b=6.96Å;Å; c=5.43 Å<br>a=b=7.01Å;Å; c=5.49 Å<br>a=b=7.02Å;Å; c=5.50 Å<br>powder	1416374	Jiang *et al.*[13]
aa-$CaCl_2$<br>aa-$CaNO_3$<br>aa-$MgNO_3$<br>aa-$MgCl_2$	Monoclinic P<br>a=5.14 Å; b=6.93 Å; c=7.61 Å; β= 100.57°<br>a=5.15 Å; b=6.97 Å; c=7.63 Å; β= 100.53°<br>powder<br>powder	652520	Bendeif and Jelsch[14]
Phe-$CaNO_3$<br>Phe-$MgNO_3$	Powder<br>powder	1012155	Ihlefeldt *et al.*[15]
Leu-$CaCl_2$<br>Leu-$CaNO_3$	Powder<br>powder	1508364	Binns *et al.*[16]

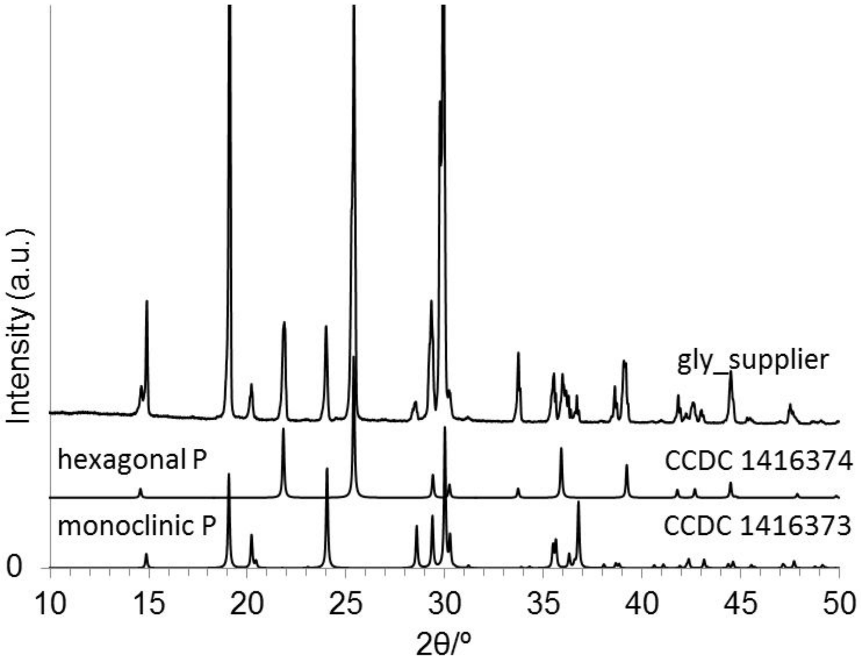

Figure S2. Comparison of the experimental X–ray powder diffraction pattern of the solid phase samples of glycine from supplier with the powder pattern calculated from the single-crystal X-ray diffraction data CCDC 1416373 (α form) and CCDC 1416374 (γ form).

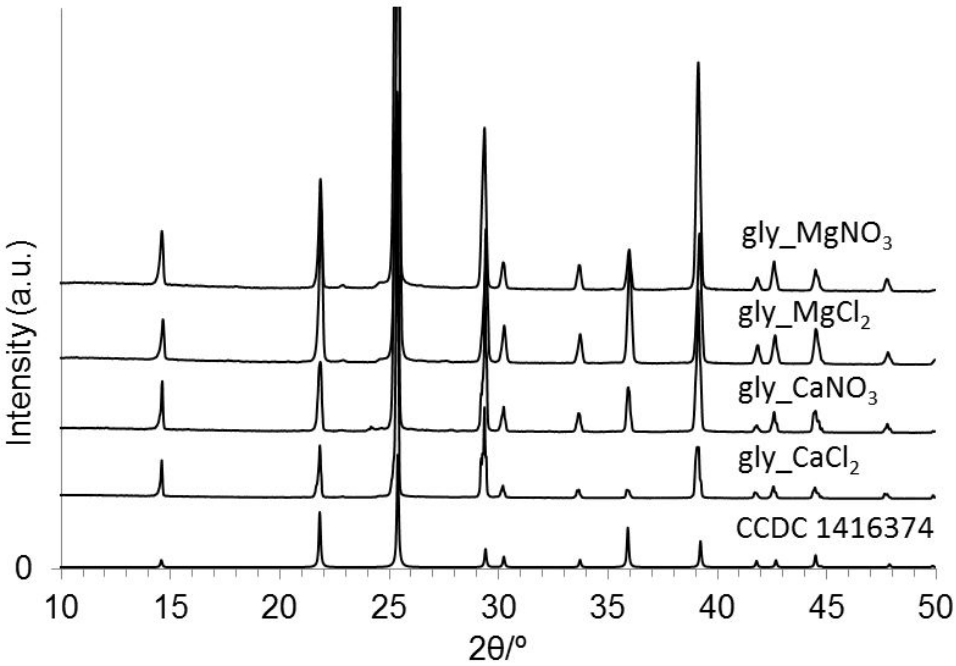

Figure S3. Comparison of the experimental X–ray powder diffraction pattern of the solid phase samples of glycine (from supplier and filtrated from aqueous solutions of chloride or nitrate salts of divalent (Mg^{2+} or Ca^{2+}) cations) with the powder pattern calculated from the single-crystal X-ray diffraction data 1416374 (γ form).

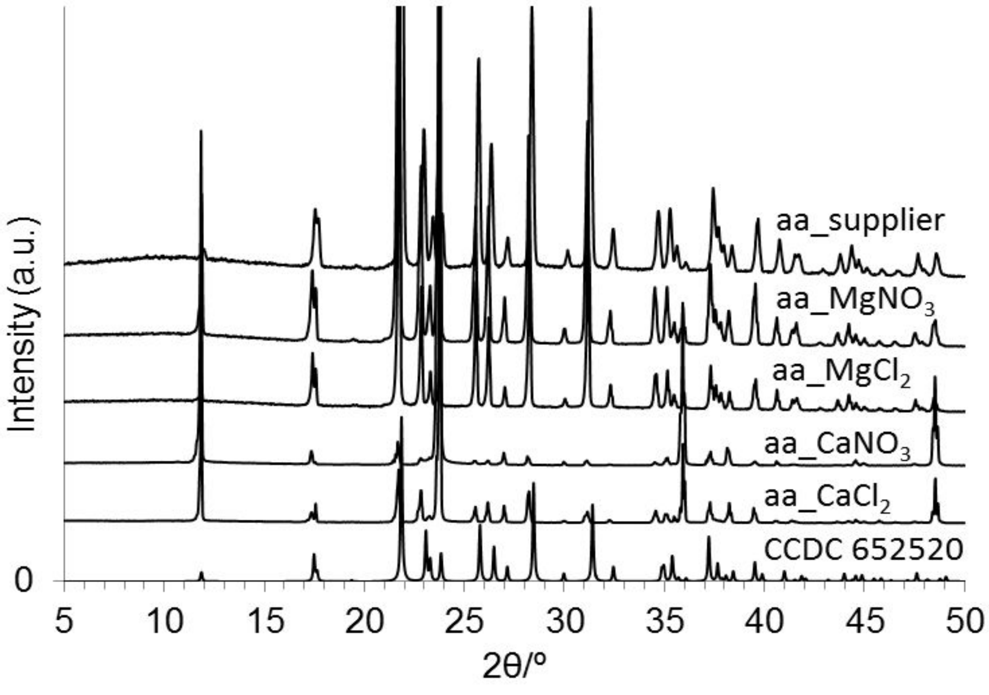

Figure S4. Comparison of the experimental X–ray powder diffraction pattern of the solid phase samples of aspartic acid (from supplier and filtrated from aqueous solutions of chloride or nitrate salts of divalent (Mg^{2+} or Ca^{2+}) cations) with the powder pattern calculated from the single-crystal X-ray diffraction data CCDC 652520.

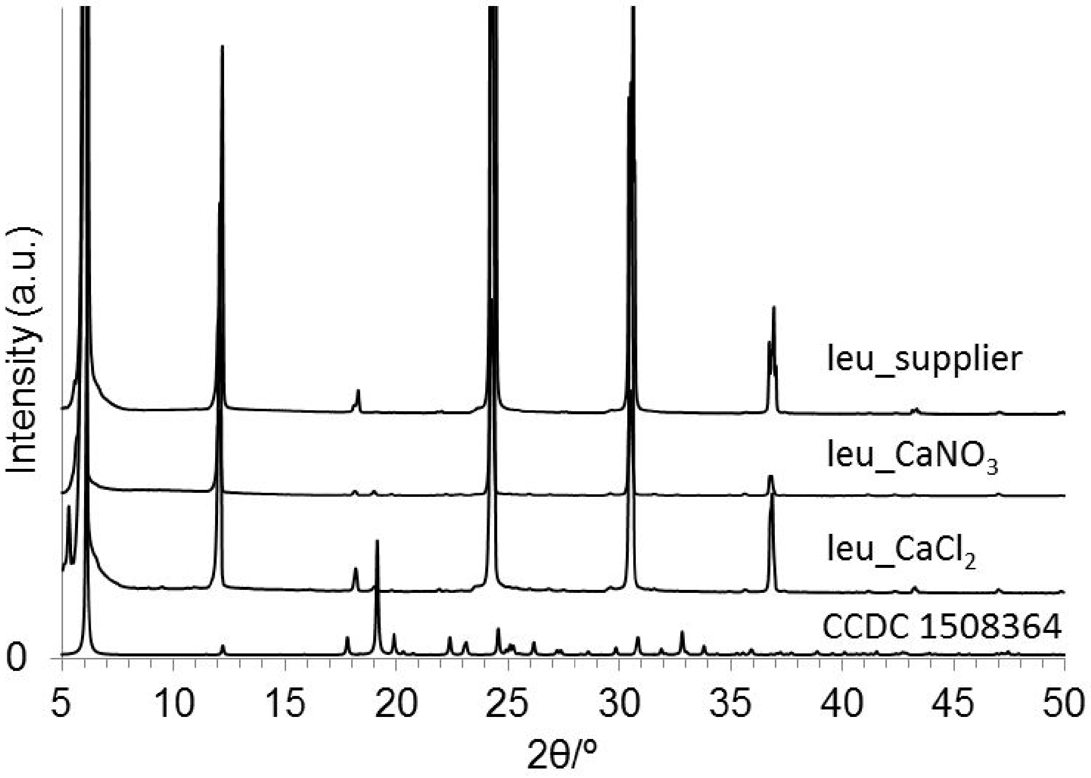

Figure S5. Comparison of the experimental X–ray powder diffraction pattern of the solid phase samples of leucine (from supplier and filtrated from aqueous solutions of calcium salts) with the powder pattern calculated from the single-crystal X-ray diffraction data CCDC 1508364.

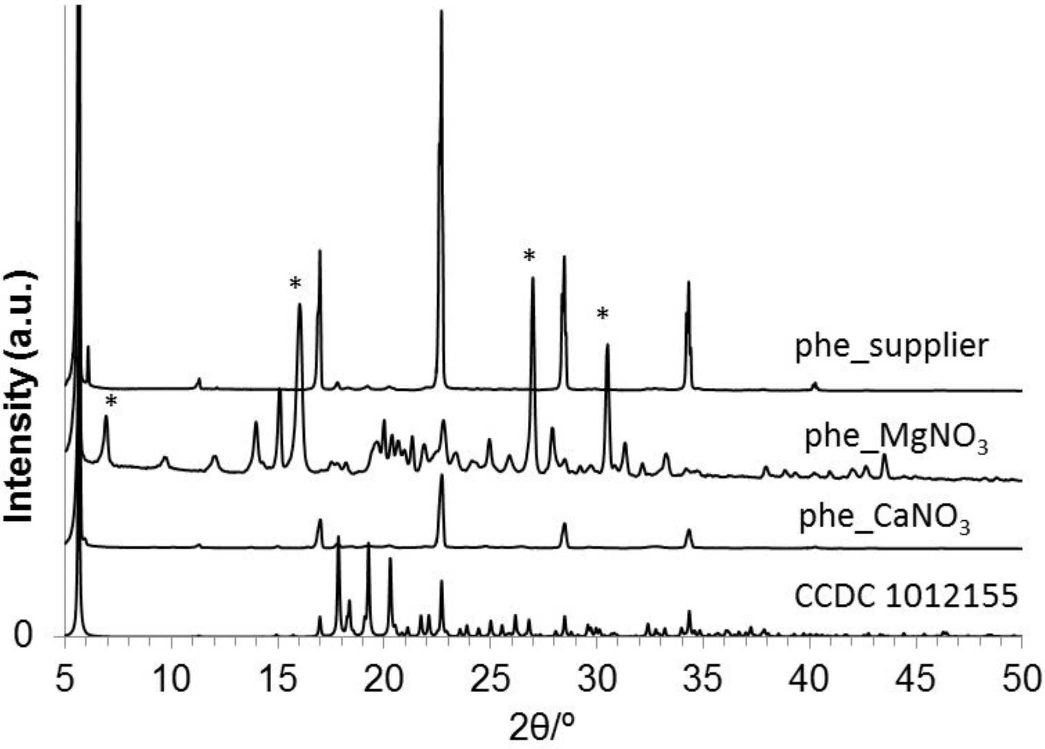

Figure S6. Comparison of the experimental X–ray powder diffraction pattern of the solid phase samples of phenylanine (from supplier and filtrated from aqueous solutions of nitrate salts of divalent (Mg^{2+} or Ca^{2+}) cations) with the powder pattern calculated from the single-crystal X-ray diffraction data CCDC 1012155 (the * denote a second phase present in the sample phe_MgNO3, searching in ICDD database version 2022 was not possible to identified this second phase).

References:

(1) Jiang, Q.; Shtukenberg, A. G.; Ward, M. D.; Hu, C. Non-Topotactic Phase Transformations in Single Crystals of β-Glycine. *Crystal Growth and Design* **2015**, *15* (6), 2568–2573.

(2) Bendeif, E.; Jelsch, C. The Experimental Library Multipolar Atom Model Refinement of L-Aspartic Acid. *Acta Crystallographica Section C: Crystal Structure Communications* **2007**, *63* (6), o361–o364.

(3) Ihlefeldt, F. S.; Pettersen, F. B.; von Bonin, A.; Zawadzka, M.; Görbitz, C. H. The Polymorphs of L-Phenylalanine. *Angewandte Chemie International Edition* **2014**, *53* (49), 13600–13604.

(4) Binns, J.; Parsons, S.; McIntyre, G. J. Accurate Hydrogen Parameters for the Amino Acid L-Leucine. *Acta Crystallographica Section B: Structural Science, Crystal Engineering and Materials* **2016**, *72* (6), 885–892.
